# Comparative effectiveness of two popular weight loss programs in women II: metabolic markers

**DOI:** 10.1186/1550-2783-8-S1-P1

**Published:** 2011-11-07

**Authors:** Mike Byrd, Michelle Mardock, Brittanie Lockard, Jonathan Oliver, Sunday Simbo, Andrew Jagim, Julie Kresta, C Baetge, Peter Jung, Majid Koozehchian, Deepesh Khanna, Mike Greenwood, Chris Rasmussen, Richard Kreider

**Affiliations:** 1 Exercise & Sport Nutrition Lab. Texas A&M University, College Station, TX 77843, USA

## Background

A number of commercial diet and exercise programs are promoted to help people lose weight and improve fitness. However, few studies have compared the effects of following different types of exercise and diet interventions on weight loss. The purpose of this study was to compare the efficacy of a more structured meal plan based diet intervention and supervised exercise program that included resistance-exercise to a traditional point based diet program with weekly counseling and encouragement to exercise.

## Methods

Fifty-one sedentary women (35±8 yrs, 163±7 cm; 90±14 kg; 47±7% body fat, 34±5 kg/m^2^) were randomized to participate in the Curves (C) or Weight Watchers (W) weight loss programs for 16-weeks. Participants in the C program were instructed to follow a 1,200 kcal/d diet for 1-week, 1,500 kcal/d diet for 3-weeks, and 2,000 kcals/d diet for 2-weeks, consisting of 30% carbohydrate, 45% protein, and 30% fat. Subjects repeated this diet for the 16-week period. Subjects also participated in the Curves circuit style resistance training program 3-days/week and were encouraged to walk at a brisk pace for 30-minutes on non-training days. This program involved performing 30-60 seconds of bi-directional hydraulic-based resistance exercise on 13 machines interspersed with 30-60 seconds of low-impact callisthenic or Zumba dance exercise. Participants in the W group followed the W point-based diet program, received weekly counseling at a local W facility, and were encouraged to increase physical activity. Fasting blood samples were obtained at 0, 4, 10, & 16 weeks and analyzed by multivariate analysis of variance (MANOVA) with repeated measures for changes in triglycerides (TG), total cholesterol (CHL), low density lipoprotein cholesterol (LDL-c), high density lipoprotein cholesterol (HDL-c), the CHL:HDL-C ratio, and blood glucose. Data are presented as percent changes from baseline for the C and W groups, respectively, after 4, 10, and 16 weeks.

## Results

MANOVA analysis of fasting lipids data revealed an overall Wilks’ Lamda significant time (p=0.001) and diet (p=0.03) effect with no significant time x diet effect (p=0.19). No significant time (p=0.72) or time x diet (p=0.36) effects were seen in changes in TG levels (C -8.0±26, -11.7±18,-2.3±26; W 4.0± 25, 5.0±32, 7.8±5 %); however, an effect of diet was seen with the C group experiencing a greater reduction in TG (p=0.06). CHL levels (p=0.001) and LDL-c levels (p=0.01) decreased in both groups over time with no differences observed between groups in changes in CHL (C -6.1±11.0, -37.9±25.8, -2.3±9.5; W -6.8±9.4, -34.2±27.4, -6.3±13.0 %, p=0.53) or LDL-c (C -6.9±17.3, -2.7±13.6, -4.6±17.2; W -5.6±14.5, -2.8±19.7, -11.4±15.9 %, p=0.16). Changes in HDL-c (C -2.1±12.5, 3.0±12.3, 5.9±18.3; W -9.5±11.5, -9.5±12.7, -1.6±14.6 %, p*_q_*=0.001) and the CHL: HDL-c ratio (C -1.8±13.1, -4.0±10.1, -3.8±12.2; W 3.4±13.4, 5.3±12.5, -3.4±14.2 %, p*_q_*=0.009) were greater in the C group. No significant time (p=0.38) or time by diet (p=0.31) effects were seen in changes in blood glucose (C -1.9±13, -0.5±12,-3.6±9; W 1.0±12, -1.0±11, 0.9±12 %).

## Conclusion

Results indicate that 16-wks of participation in the C and W programs promoted improvements in CHL and LDL-c. However, adherence to a more structured meal plan based diet and supervised exercise program promoted more favorable changes in TG, HDL-c and the ratio of CHL: HDL-c.

## Funding

Supported by Curves International (Waco, TX)

